# Supplementary material for: Cross-species metabolomic analysis of tau- and DDT-related toxicity
Source: PNAS Nexus. 2022 May 3;1(2):pgac050. doi: 10.1093/pnasnexus/pgac050 (PMC9186048; doi:10.1093/pnasnexus/pgac050)
Supplement: pgac050_Supplemental_File [file pgac050_supplemental_file.docx]

Supplemental tables and figures associated with: **Cross-species metabolomic analysis of tau- and DDT-related toxicity**

**Figure S1.** The feature putatively identified as a metabolite of DDT was detected in more people with Alzheimer’s disease (A) and had higher levels in people with Alzheimer’s disease as well (B). There was no correlation evident between pTau181 and the DDT metabolite (C). In (D and F), a Manhattan plot shows features significantly associated with the metabolite in human plasma (D) and CSF (F). These features enriched several pathways (E and G) related to inflammation and amino acid metabolism.


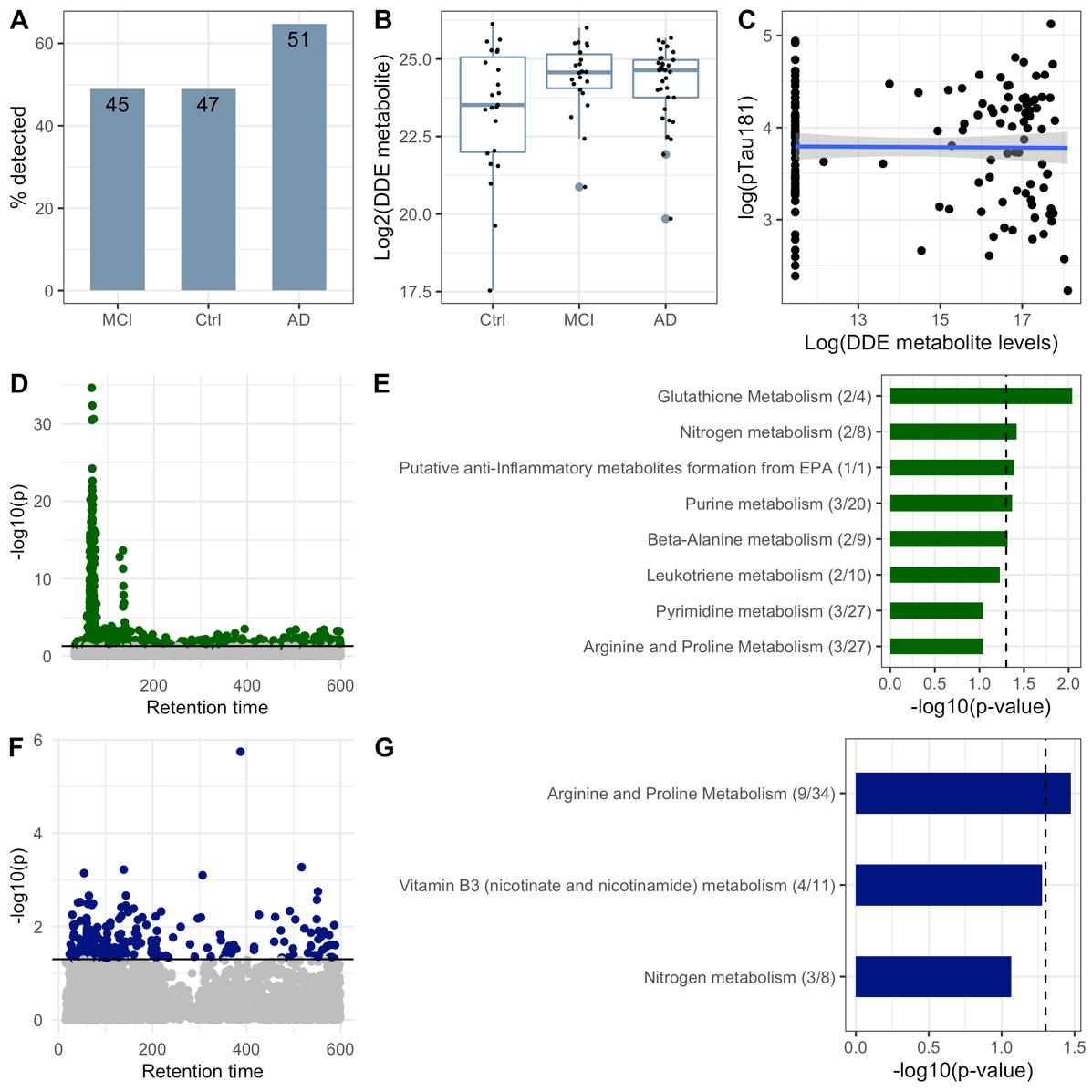


**Figure S2.** Six different swim behaviors measured in wildtype, aggregating, and non-aggregating worms exposed to DDT including asymmetry, attenuation, brush stroke, reverse swim, stretch and body wave number. The aggregating strain spent more time swimming in reverse than the other strains or treatments. The aggregating strain also has a lower brush stroke compared to the wildtype worms. None of the other swim behaviors were significantly different between the different strains or treatments.


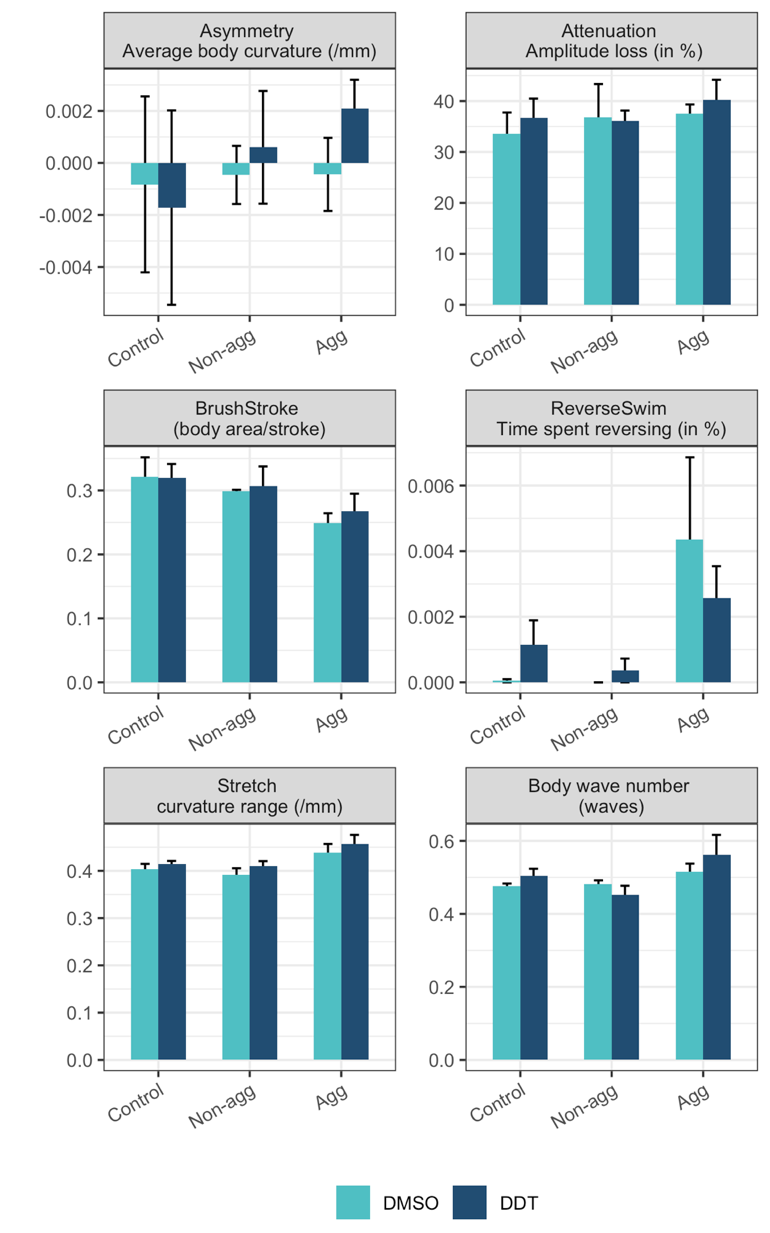


**Figure S3.** The heatmap shows annotations and clustering of features measured using the HILIC column under negative electron spray ionization.


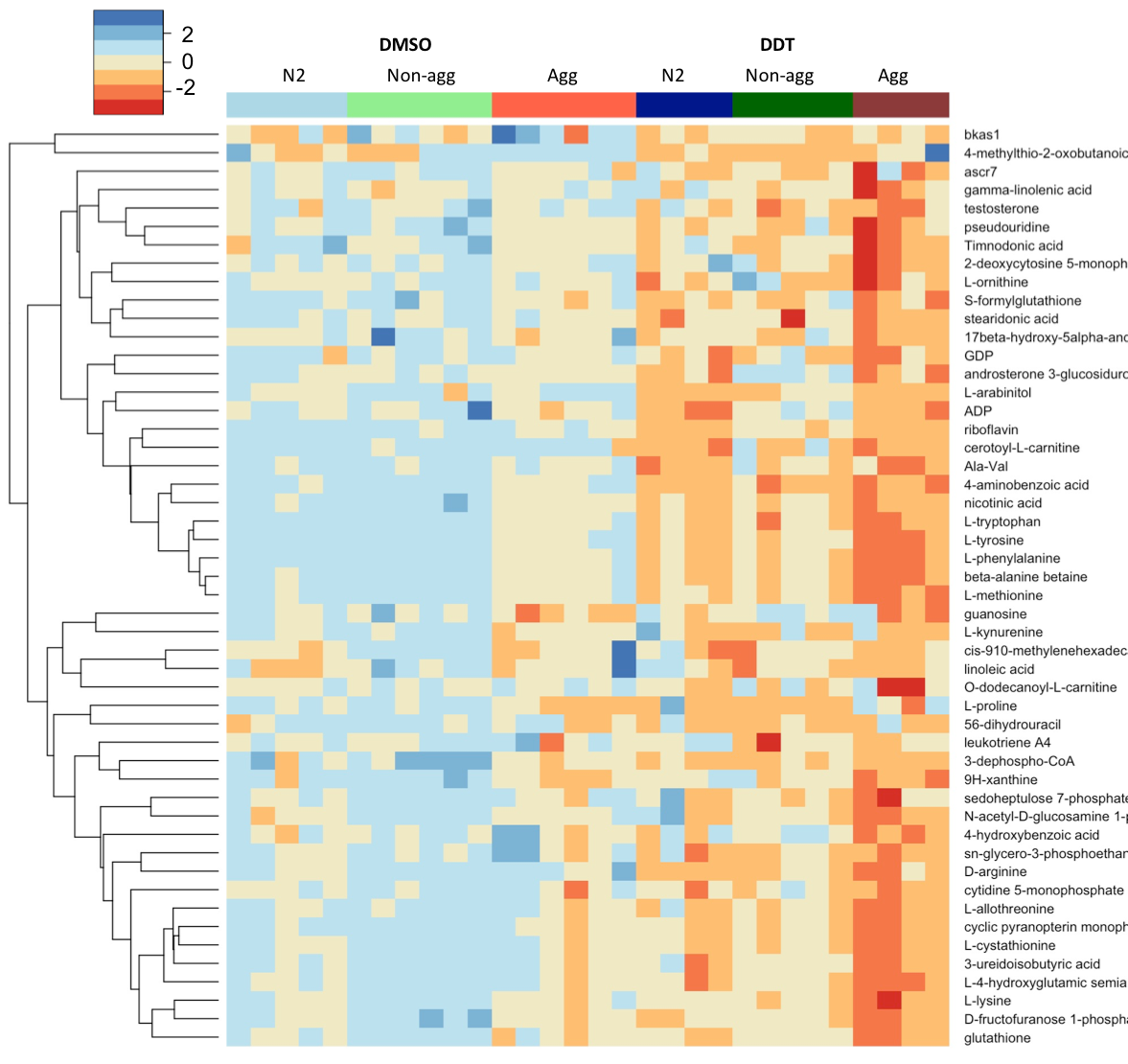


**Figure S4.** The heatmap shows annotations and clustering of features measured using the C18 column under positive electron spray ionization.


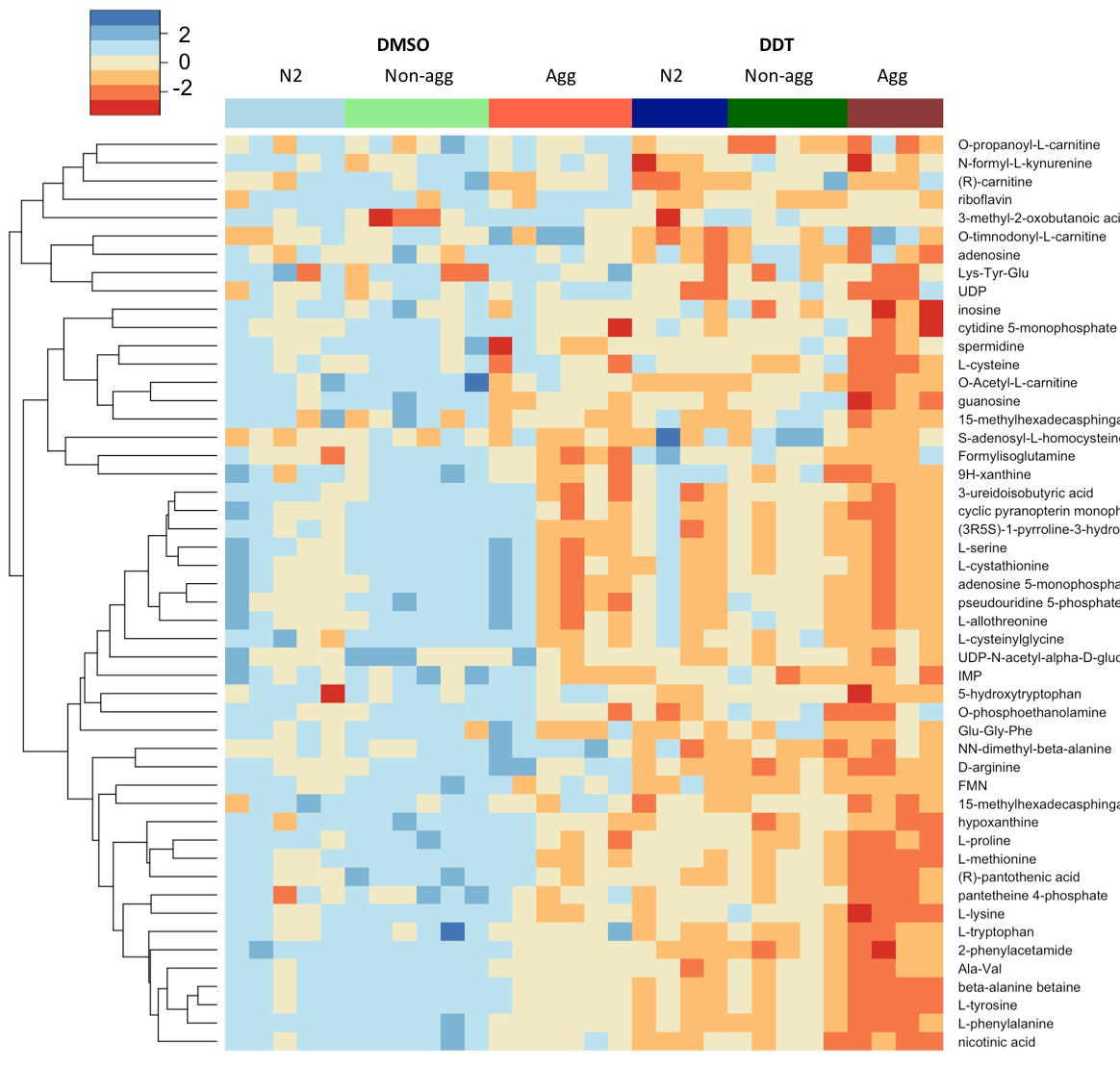


**Figure S5.** The heatmap shows annotations and clustering of features measured using the C18 column under negative electron spray ionization.


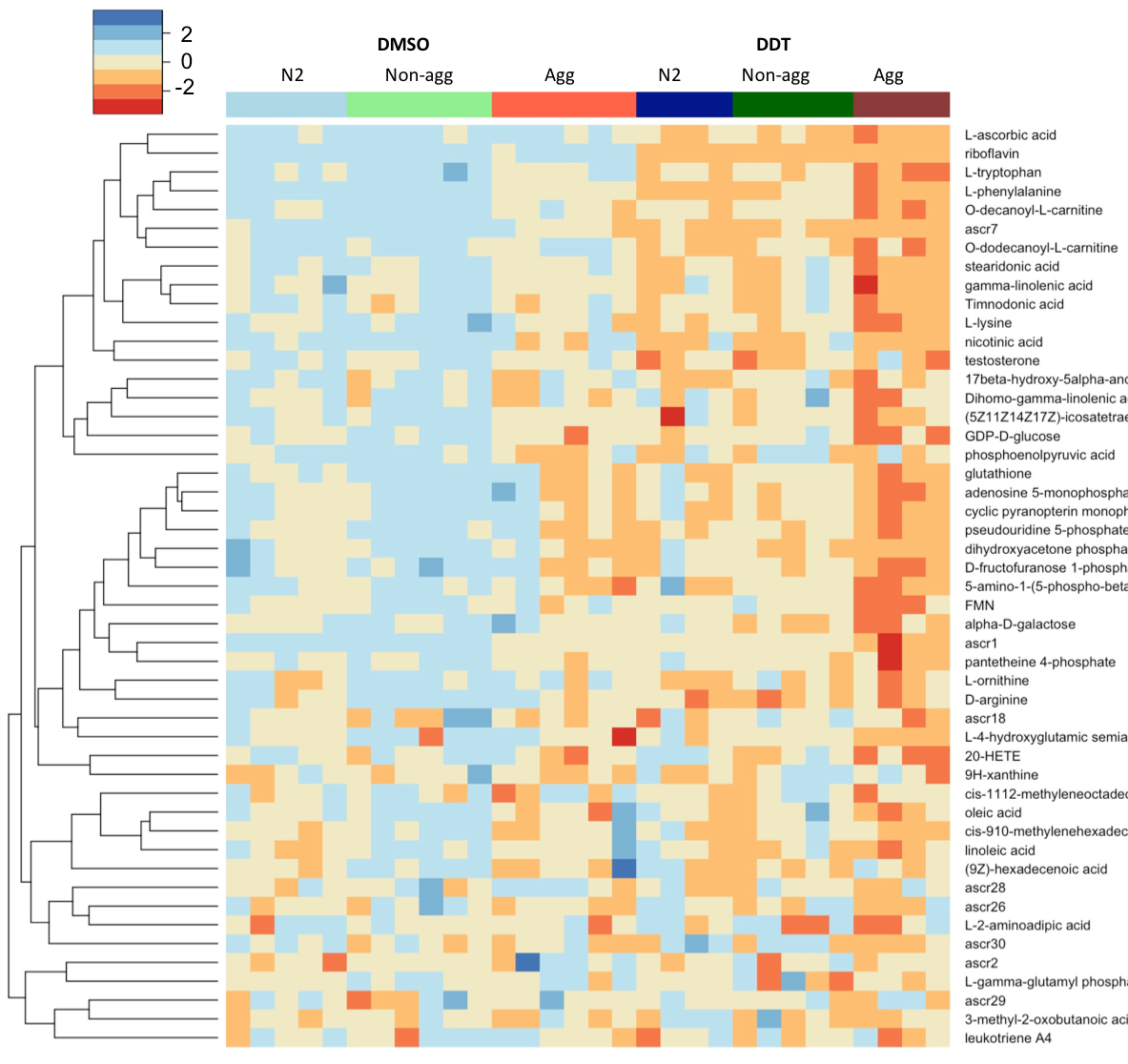


**Figure S6.** These plots indicate the presence of interaction between treatment and strain (BR5270: pro-aggregating, BR5271: non-aggregating, N2: wildtype) as assessed through two-way ANOVAs.

**Table S1.** Retention time and ions monitored to quantify and confirm DDT metabolites

| Analyte | Retention Time  (min) | Quantifying Ion  (m/z) | Confirming Ion 1  (m/z) | Confirming Ion 2  (m/z) | LOD  (ppb) |
| --- | --- | --- | --- | --- | --- |
| o,p'-DDE | 11.13 | 245.9999 | 247.9968 | 317.9345 | 0.016 |
| o,p'-DDT | 12.80 | 235.0076 | 165.0699 | 237.0047 | 0.010 |
| p,p'-DDE | 11.70 | 245.9999 | 247.9968 | 317.9345 | 0.029 |
| p,p'-DDT | 13.32 | 235.0076 | 165.0699 | 237.0047 | 0.029 |
| 4,4'-DDE (^13^C_12_) | 11.70 | 260.0370 | 188.1021 | 258.0400 |  |
| 4,4'-DDT (D_8_) | 13.27 | 243.0576 | 173.1200 | 245.0549 |  |

**Table S2.** Metabolites similarly associated with DDT exposure or it’s putative metabolite in *C. elegans* and human plasma and human CSF.

| **Species** | **Metabolite name** | **Direction of association** | **p.value** | **KEGG ID** | **mz** | **time** | **Adduct matched** | **Mass.Diff** | **ESI** |
| --- | --- | --- | --- | --- | --- | --- | --- | --- | --- |
| ***C. elegans* and Human plasma** | | | | | | | | | |
| *C. elegans* | Tryptophan | negative | 1.75E-07 | C00078 | 205.097166 | 180.083376 | M+H[1+] | 0.000012 | positive |
| Human plasma | Tryptophan | negative | 0.00388314 | C00078 | 159.091815 | 84.5006925 | M-HCOOH+H[1+] | 0.000039 | positive |
| *C. elegans* | Phenylalanine | negative | 3.26E-08 | C00079 | 166.08637 | 24.5254799 | M+H[1+] | 0.000114 | positive |
| Human plasma | Phenylalanine | negative | 0.04689407 | C00079 | 149.059875 | 89.1749268 | M-NH3+H[1+] | 0.000099 | positive |
| Human plasma | Tyrosine | negative | 0.01127123 | C00082 | 146.060122 | 85.9253095 | M-H4O2+H[1+] | 0.000146 | positive |
| *C. elegans* | Tyrosine | negative | 1.94E-06 | C00082 | 200.091371 | 187.818882 | M+H2O+H[1+] | 0.000399 | positive |
| *C. elegans* | Leucine | negative | 4.41E-07 | C00123 | 132.101866 | 179.945364 | M+H[1+] | 0.000039 | positive |
| Human plasma | Leucine | negative | 0.00290084 | C00123 | 133.10536 | 122.308125 | M(C13)+H[1+] | 0.000084 | positive |
| *C. elegans* | Threonine | negative | 1.50E-04 | C00188 | 118.051016 | 199.786799 | M-H[-] | 0.000050 | negative |
| Human plasma | Threonine | negative | 4.16E-04 | C00188 | 121.069079 | 131.275071 | M(C13)+H[1+] | 0.000203 | positive |
| *C. elegans* | Isoleucine | negative | 4.41E-07 | C00407 | 132.101866 | 179.945364 | M+H[1+] | 0.000039 | positive |
| Human plasma | Isoleucine | negative | 0.00290084 | C00407 | 133.10536 | 122.308125 | M(C13)+H[1+] | 0.000084 | positive |
| Human plasma | Serotonin | negative | 0.00388314 | C00780 | 159.091815 | 84.5006925 | M-H2O+H[1+] | 0.000139 | positive |
| *C. elegans* | Serotonin | negative | 1.28E-05 | C00780 | 199.084645 | 187.885234 | M+Na[1+] | 0.000405 | positive |
| Human plasma | (15S)-15-Hydroxy-5,8,11-cis-13-trans-eicosatetraenoate | positive | 0.01164717 | C04742 | 303.231822 | 105.180982 | M-H2O+H[1+] | 0.000045 | positive |
| *C. elegans* | (15S)-15-Hydroxy-5,8,11-cis-13-trans-eicosatetraenoate | positive | 0.0043616 | C04742 | 319.227701 | 33.7918996 | M-H[-] | 0.000167 | negative |
| *C. elegans* | 5(S)-HETE | positive | 0.0043616 | C04805 | 319.227701 | 33.7918996 | M-H[-] | 0.000167 | negative |
| Human plasma | 5(S)-HETE | positive | 0.01164717 | C04805 | 303.231822 | 105.180982 | M-H2O+H[1+] | 0.000045 | positive |
| *C. elegans* | Allothreonine | negative | 1.50E-04 | C05519 | 118.051016 | 199.786799 | M-H[-] | 0.000050 | negative |
| Human plasma | Allothreonine | negative | 4.16E-04 | C05519 | 121.069079 | 131.275071 | M(C13)+H[1+] | 0.000203 | positive |
| *C. elegans* | Gentisate aldehyde | positive | 1.88E-06 | C05585 | 137.024463 | 111.340328 | M-H[-] | 0.000045 | negative |
| Human plasma | Gentisate aldehyde | positive | 0.04186037 | C05585 | 139.039056 | 319.679094 | M+H[1+] | 0.000080 | positive |
| Human plasma | (9Z,12Z,15Z)-Octadecatrienoic acid | positive | 0.02329459 | C06427 | 279.231785 | 101.226519 | M+H[1+] | 0.000091 | positive |
| *C. elegans* | (9Z,12Z,15Z)-Octadecatrienoic acid | positive | 0.0092333 | C06427 | 277.217187 | 31.8480979 | M-H[-] | 0.000117 | negative |
| ***C. elegans* and Human cerebrospinal fluid** | | | | | | | | | |
| Human CSF | Serine | negative | 0.03913253 | C00065 | 88.0396415 | 160.280173 | M-H2O+H[1+] | 3.65E-04 | positive |
| *C. elegans* | Serine | negative | 0.012551 | C00065 | 145.061874 | 202.377695 | M+ACN-H[-] | 1.28E-05 | negative |
| *C. elegans* | Ornithine | negative | 1.65E-04 | C00077 | 116.070629 | 20.8902467 | M-NH3+H[1+] | 2.54E-05 | positive |
| Human CSF | Ornithine | negative | 0.00303989 | C00077 | 133.097198 | 41.242106 | M+H[1+] | 2.16E-05 | positive |
| Human CSF | Anthranilate | negative | 0.02293147 | C00108 | 94.0654803 | 206.098432 | M-CO2+H[1+] | 3.04E-04 | positive |
| *C. elegans* | Anthranilate | negative | 4.38E-08 | C00108 | 136.040469 | 44.3311943 | M-H[-] | 6.71E-05 | negative |
| *C. elegans* | Tyramine | negative | 8.53E-08 | C00483 | 120.080788 | 176.658188 | M-H2O+H[1+] | 4.76E-05 | positive |
| Human CSF | Tyramine | negative | 0.02238869 | C00483 | 138.09117 | 167.458773 | M+H[1+] | 2.06E-04 | positive |
| *C. elegans* | Ornithine | negative | 1.65E-04 | C00515 | 116.070629 | 20.8902467 | M-NH3+H[1+] | 2.54E-05 | positive |
| Human CSF | Ornithine | negative | 0.00303989 | C00515 | 133.097198 | 41.242106 | M+H[1+] | 2.16E-05 | positive |
